# Understanding the Enzyme (*S*)‑Norcoclaurine Synthase Promiscuity to Aldehydes and Ketones

**DOI:** 10.1021/acs.jcim.3c01773

**Published:** 2024-05-22

**Authors:** Brunno A. Salvatti, Marcelo A. Chagas, Phillipe O. Fernandes, Yan F. X. Ladeira, Aline S. Bozzi, Veronica S. Valadares, Ana Paula Valente, Amanda S. de Miranda, Willian R. Rocha, Vinicius G. Maltarollo, Adolfo H. Moraes

**Affiliations:** † Departamento de Química, Instituto de Ciências Exatas, Universidade Federal de Minas Gerais, Belo Horizonte 31270-901, Brazil; ‡ Departamento de Ciências Exatas, 133635 Universidade do Estado de Minas Gerais, João Monlevade, Minas Gerais 35930-314, Brazil; § Departamento de Produtos Farmacêuticos, Faculdade de Farmácia, Universidade Federal de Minas Gerais, Belo Horizonte 31270-901, Brazil; ∥ Departamento de Bioquímica e Imunologia, Instituto de Ciências Biológicas, Universidade Federal de Minas Gerais, Belo Horizonte 31270-901, Brazil; ⊥ Centro Nacional de Ressonância Magnética Nuclear, Instituto de Bioquímica Médica Leopoldo de Meis, Centro de Ciências da Saúde, 28125 Universidade Federal do Rio de Janeiro, Rio de Janeiro 21.941-902, Brazil

## Abstract

The (*S*)-norcoclaurine synthase from (*Tf*NCS) stereoselectively catalyzes the Pictet–Spengler reaction between dopamine and 4-hydroxyphenylacetaldehyde to give (*S*)-norcoclaurine. *Tf*NCS can catalyze the Pictet–Spengler reaction with various aldehydes and ketones, leading to diverse tetrahydroisoquinolines. This substrate promiscuity positions *Tf*NCS as a highly promising enzyme for synthesizing fine chemicals. Understanding carbonyl-containing substrates’ structural and electronic signatures that influence *Tf*NCS activity can help expand its applications in the synthesis of different compounds and aid in protein optimization strategies. In this study, we investigated the influence of the molecular properties of aldehydes and ketones on their reactivity in the *Tf*NCS-catalyzed Pictet–Spengler reaction. Initially, we compiled a library of reactive and unreactive compounds from previous publications. We also performed enzymatic assays using nuclear magnetic resonance to identify some reactive and unreactive carbonyl compounds, which were then included in the library. Subsequently, we employed QSAR and DFT calculations to establish correlations between substrate-candidate structures and reactivity. Our findings highlight correlations of structural and stereoelectronic features, including the electrophilicity of the carbonyl group, to the reactivity of aldehydes and ketones toward the *Tf*NCS-catalyzed Pictet–Spengler reaction. Interestingly, experimental data of seven compounds out of fifty-three did not correlate with the electrophilicity of the carbonyl group. For these seven compounds, we identified unfavorable interactions between them and the *Tf*NCS. Our results demonstrate the applications of *in silico* techniques in understanding enzyme promiscuity and specificity, with a particular emphasis on machine learning methodologies, DFT electronic structure calculations, and molecular dynamic (MD) simulations.

## Graphical abstract

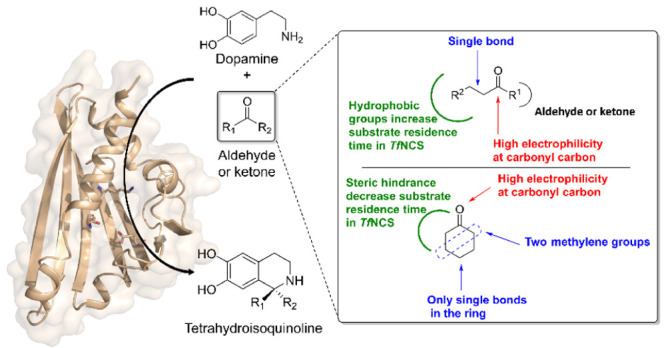

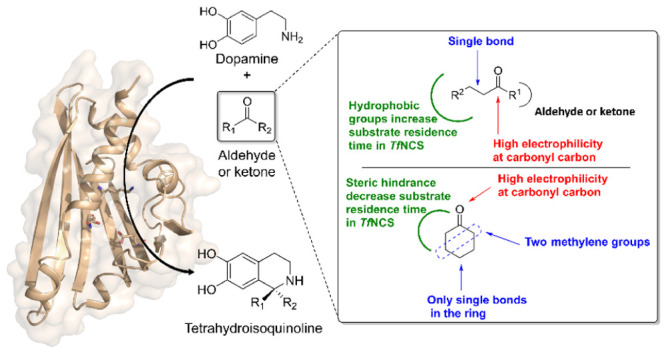

## Introduction

Enzymes are ancient catalysts whose functions have evolved under biological, physical, and chemical pressure. Their evolutionary processes are essential to understanding how life has evolved on our planet. Furthermore, enzymes have also gained prominence in practical applications as biocatalysts.^1^ Enzymes have advantages over traditional catalysts: they can catalyze chemical reactions efficiently under mild and eco-friendly conditions; they are nontoxic and biodegradable, obtained from renewable sources, and show high chemo-, regio-, and stereoselectivity. Using enzymes as biocatalysts can reduce the chemical synthesis steps, simplify product purification, provide cleaner reactions, and decrease costs and waste.^2^ Over the years, the chemical, pharmaceutical, medical, oil, biofuel, pump, and textile industries have used enzymes to obtain specific products. The global market share of industrial enzymes is estimated at US $7.1 billion, with an annual growth rate of around 8%.^3^

Enzymes have been increasingly used as catalysts in organic synthesis. In the last four decades, advances in areas such as protein engineering and molecular biology have provided tools to overcome drawbacks usually associated with biocatalysts, such as limited availability, low stability, and narrow substrate scope, thus allowing for biocatalyzed reactions with non-natural substrates under non-natural conditions employing modified enzymes.^2,4,5^ Biocatalysis is, however, still a developing field. In recent days, the integration of advanced molecular genetics, metagenomics, and bioinformatics is expected to play an essential role in the discovery and development of novel enzymes and novel enzyme functions.^2^

Among the most important advantages of enzymes in organic synthesis over nonenzymatic catalysts is their high selectivity. Therefore, their use can shorten synthetic routes, simplify product purification, provide cleaner reactions, and decrease costs and waste.^2^ Moreover, the high chemo-, regio-, and stereoselectivity displayed by biocatalysts makes them particularly suitable for synthesizing bioactive compounds, including drugs, which are often highly functionalized compounds with one or more chirality centers. For instance, enzymes such as lipases, oxidoreductases, and transaminases have been used in industrial processes, and an increasing number of enzymes have been applied in the synthesis of fine chemicals in the academic arena.^6^ In addition, biocatalysts have also been considered promising tools in the early stages of drug discovery and development since they may enable better exploitation of the chemical space by giving access to compounds challenging to synthesize using nonenzymatic catalysis.^7–9^

The enzymes (*S*)-norcoclaurine synthases ((*S*)-NCSs) have emerged as promising biocatalysts for the synthesis of chiral tetrahydroisoquinolines.^10–21^ These enzymes can catalyze the Pictet–Spengler reaction, thus being classified as Pictet–Spenglerases. The (*S*)-norcoclaurine synthases can be found in angiosperms such as ,^22^ ,^23^ and .^24^ These enzymes are involved in the biosynthesis of many essential alkaloids with diverse pharmacological activities, such as morphine, berberine, and others.^24^ The (*S*)-norcoclaurine synthase from , *Tf*NCS, takes part in the enantioselective catalysis of a Pictet–Spengler condensation reaction between dopamine and the aldehyde 4-hydroxyphenylacetaldehyde, producing (*S*)-norcoclaurine (Figure 1a). Importantly, (*S*)-norcoclaurine is a key intermediate for the synthesis of alkaloid pain killers such as morphine and codeine. The obtaining of these alkaloids still relies on supplies from opium poppy crops,^25^ thus reflecting the need for suitable synthetic methodologies toward enantiopure tetrahydroisoquinolines.

**Figure 1:**
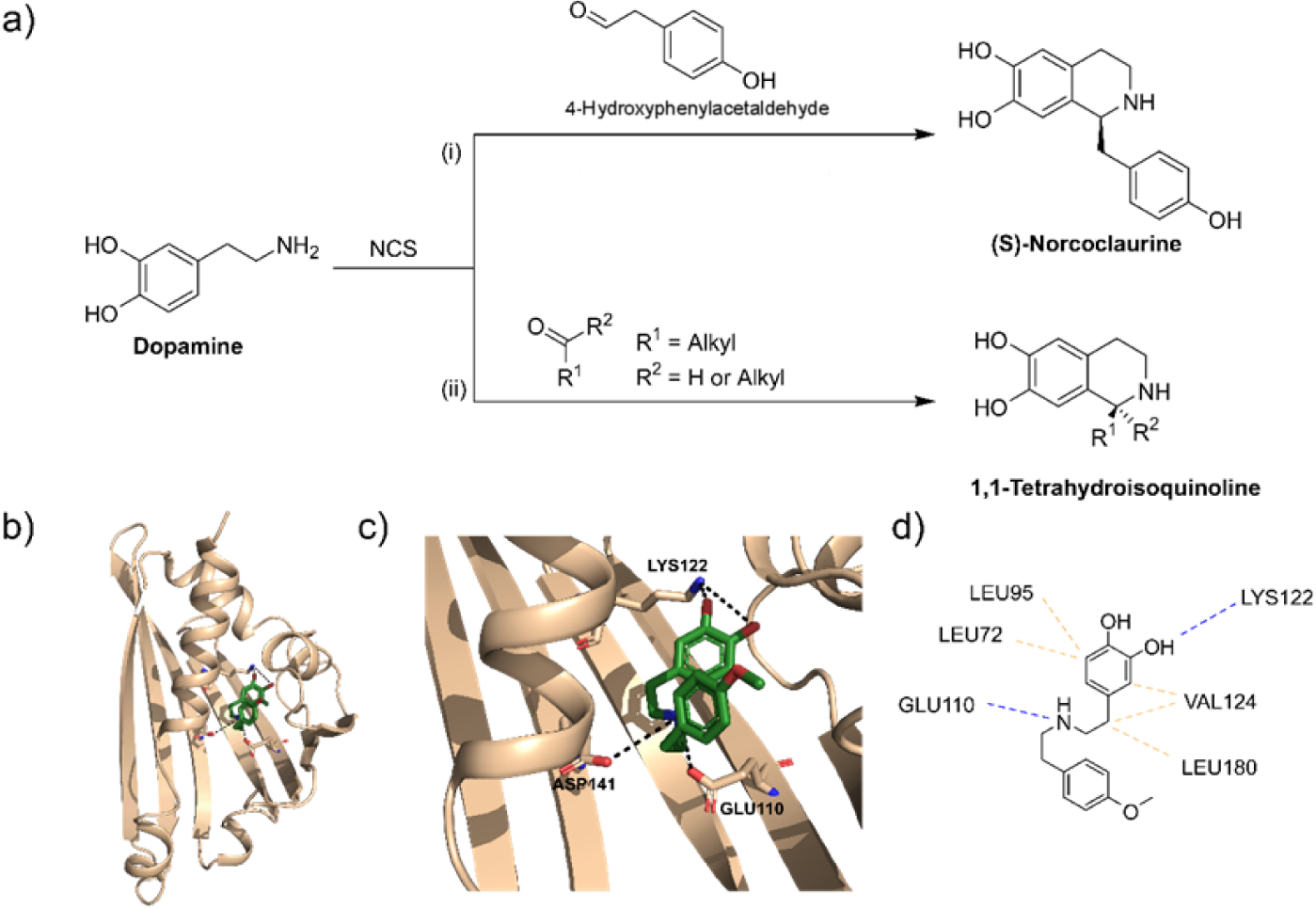
(a) *Tf*NCS-catalyzed Pictet–Spengler reaction to give (i) (*S*)-norcoclaurine and (ii) tetrahydroisoquinoline from aldehydes and ketones; (b) T*f*NCS crystal structure bound to a transition-state mimetic compound (pdb id: 5NON); (c) *Tf*NCS catalytic site: side chains of residues Glu110, Lys122, and Asp141 are shown as well as the structure of the transition-state mimetic compound colored in tan (pdb id: 5NON); (d) interactions between *Tf*NCS residues and the transition-state mimetic compound identified by using the software Discovery Studio.

In addition to the natural carbonyl substrate, *Tf*NCS can catalyze the condensation reaction with several aldehydes, producing various tetrahydroisoquinolines^20,26,27^ (Figure 1a). Lichman et al.^15^ showed that *Tf*NCS was able to catalyze the Pictet–Spengler reaction with ketones to produce 1,1-tetrahydroisoquinolines (Figure 1a). Noteworthily, nonenzymatic enantioselective Pictet–Spengler reactions have been scarcely reported.^28–31^ The synthesis of enantiopure 1,1-tetrahydroisoquinolines by the nonenzymatic Pictet–Spengler reaction has not been reported. *Tf*NCS’s promiscuity toward aldehydes and ketones is an essential property of this enzyme since it could lead to different applications in the fine chemical industry.^20^ Because tetrahydroisoquinolines have been reported as a useful scaffold in medicinal chemistry,^32,33^ understanding and expanding the substrate scope of *Tf*NCS would allow for full exploitation of this enzyme as a valuable tool in the synthesis of compound series for drug discovery and development programs.

*Tf*NCS belongs to the Bet v 1-like structural superfamily of proteins, which comprises the PR-10 pathogen’s proteins as Bet v 1,^34–36^ Fag s 1,^37,38^ other enzymes, and small organic compound carriers found in many organisms.^24,36,39–41^ The *Tf*NCS structure comprises seven beta strands and four alpha helices folded into alpha–beta sandwich fold^22,35^ (Figure 1b). The *Tf*NCS catalytic site (Figure 1c,d) is composed of amino acid residues Lys122, Glu110, and Asp141. Other residues play an essential role in *Tf*NCS catalytic activity by interacting with substrates and reaction intermediates or modulating the electronic environment. Lichman et al.^22^ have identified the *Tf*NCS residues directly involved in binding dopamine and aldehyde. They solved the crystal structure of the *Tf*NCS bound to a compound analog to a reaction transition state (Figure 1b–d). Besides the catalytic site residues, Tyr108 and Met183 are necessary for the substrate binding and orientation.^22,26^ They also determined dopamine and carbonyl substrate orientations inside the cavity that corroborate the “dopamine-first mechanism.” According to this proposed mechanism, binding of *Tf*NCS to dopamine is followed by the interaction with the carbonyl counterpart (an aldehyde or ketone). Sheng and Himo^42^ have conducted quantum mechanical calculations employing a cluster model, proposed a detailed dopamine-first mechanism, and used the correlation between energy and stereoselectivity to explain the enzymatic enantioselectivity.

*In silico* studies have contributed to the detailed atomic characterization of many enzyme reactions’ mechanisms.^43^ Machine learning-based strategies have been applied in many areas, such as protein structure modeling from the primary sequence,^44^ design of new drug candidates,^45^ and understanding of enzyme properties.^46^ However, few studies have been performed to understand the promiscuity and specificity of enzymes toward substrates. A better understanding of carbonyl compounds’ molecular properties and structural features that correlate with their acceptance as a substrate by *Tf*NCS could help to understand (*S*)-NCS specificity and guide site-directed mutagenesis for enzyme optimization. In this work, we used QSAR^47^ and DFT^48^ calculations to identify correlations between substrate-candidate structures and *Tf*NCS acceptance. We investigated fundamental intermolecular interactions that stabilize *Tf*NCS:substrate complexes with molecular dynamics simulation.^49^ The structure of the carbonyl compounds plays a significant role in classifying a compound as a substrate, i.e., a ketone or aldehyde that has been found reactive in the (*S*)-NCS-catalyzed reaction to give the corresponding tetrahydroisoquinoline. Interestingly, for 7 out of 53 compounds, the experimental reactivity in the *Tf*NCS-catalyzed Pictet–Spengler reaction could not be correlated to the electrophilicity of the carbonyl group. For these outliers, we found unfavorable interactions between the enzyme and the substrate candidates through molecular modeling. Together, our results comprise studies describing a set of *in silico* techniques to understand enzyme promiscuity and specificity, with particular attention paid to the combination of machine learning methodologies, density functional theory (DFT) calculations, and molecular dynamic (MD) simulation.

## Material and Methods

### *Tf*NCS Substrate Library Organization

The *Tf*NCS substrate library was organized with compounds evaluated in *Tf*NCS activity assays using similar experimental protocols. 49 carbonyl compounds, including aldehydes and ketones, were selected to build a *Tf*NCS carbonyl substrate library. Additional 4 compounds were added by monitoring the enzymatic reaction via NMR spectroscopy. The list of 53 carbonyl compounds that compose the library, their chemical structures, and their identifier numbers are shown in Figures S01, S02, and S03. The carbonyl substrate candidates were classified as reactive if they were found to react with dopamine in *Tf*NCS-catalyzed reactions or as unreactive if no reaction was observed.

### *Tf*NCS Expression and Purification

The sequence of N-terminally His6-tagged Δ19*Tf*NCS, coding for residues 19–210 of the whole (*S*)- norcoclaurine synthase from (UniProt accession code: Q67A25), was commercially synthesized and cloned by GenScript (Piscataway, USA) in a pET28a plasmid. BL21 (DE3) cells were transformed with the plasmid and cultivated at 37 °C in 100 mL of Luria–Bertani (LB) medium with 50 μg mL^–1^ kanamycin for 16 h under shaking at 180 rpm. Cells were harvested by centrifugation and used to inoculate 900 mL of LB. Protein expression was induced with 0.5 mM isopropyl-β-d-thiogalactopyranoside (IPTG) when the OD at 600 nm was between 0.6 and 0.8. Cultures were incubated for 16 h at 22 °C under shaking at 180 rpm. Cells were centrifuged for 40 min at 4 °C and 4000 rpm in a Thermo Scientific TX-750 Swinging Bucket Rotor (Thermo Fisher, USA). Harvested cells were resuspended in 50 mM sodium phosphate at pH 7.0, 10 mM imidazole, and 2 mM 1,4-dithiothreitol (DTT). The cell suspension was frozen at −20 °C, thawed at room temperature with 3 mg mL^–1^ lysozyme and 2 mM phenylmethylsulfonyl fluoride (PMSF), and passed 10× through a 0.55 mm gauge needle for cell disruption. The lysate was centrifuged at 11 000 rpm for 40 min at 4 °C. Δ19*Tf*NCS was purified from the soluble fraction by Ni^2+^ affinity chromatography using a HisTrap HP Ni-NTA column (GE Healthcare, USA) in 50 mM sodium phosphate pH 7.0, containing 10 mM imidazole and 2 mM DTT, and eluted with an imidazole gradient up to 500 mM. Fractions of Δ19*Tf*NCS were then purified with molecular size exclusion chromatography using a Superdex 75 10/300 GL column (GE Healthcare, USA) previously equilibrated in 50 mM HEPES pH 7.0, 100 mM NaCl, and 2 mM DTT. The purified protein was stored at −20 °C for further use.

### NMR-Based Monitoring of Enzymatic Reactions

The Pictet–Spengler reaction of dopamine with hexanal catalyzed by the heterologous expression of *Tf*NCS was carried out following previously described procedures^15,20^ and monitored by ^1^H NMR. The reaction was conducted in 50 mM HEPES buffer solution, 5 mM ascorbic acid, 2.5 mM aldehyde, 2.5 mM dopamine, 10% v/v DMSO, 10% v/v D_2_O, and 0.139 mM DSS, as an internal NMR standard, at 37 °C. A control reaction was also carried out in the absence of the enzyme. Consumption of dopamine and hexanal and the production of the expected tetrahydroisoquinoline were followed by monitoring of the ^1^H NMR signals. The NMR assignments were performed based on the study by Lichman et al.^26^ who previously purified and solved the structure of the tetrahydroquinoline produced in the *Tf*NCS Pictet–Spengler catalyzed reaction between dopamine and hexanal by NMR spectroscopy. The reaction was evaluated in three different moments after 5 min, 2 h, and 24 h after the *Tf*NCS addition. The ^1^H NMR experiments were acquired using the ^1^H solvent-presaturation NOESY sequence (Bruker code: noesypr1d) with the following acquisition parameters: number of scans = 128, number of acquisition points = 64k, recovery time = 30 s, and mixing time = 10 ms. The ^1^H NMR spectra were processed and analyzed by using Topspin version 4.1.0 software (Bruker BioSpin, USA). The NMR spectra were processed using zero felling with 2-fold the number of acquisition points and line broadening of 0.3 Hz.

### Three-Dimension Structure Modeling of Substrate Candidates

The three-dimensional structures of *Tf*NCS substrate candidates were sketched using the software ChemSketch^50^ and modeled using the server CACTUS (https://cactus.nci.nih.gov ). The 3D chemical structures were optimized in a vacuum with the software AVOGADRO,^51,52^ using the Universal Force Field.^53^

### QSAR Model Construction

The carbonyl substrate candidate’s ionization state was attributed at pH 7.4 using the *fixpka* option in the QUACPAC software.^54^ The minimum energy conformer of each compound was identified by conformational analysis in the software OMEGA^55^ using the MMFF94 force field. The data set was divided into training and test sets with 80 and 20%, respectively, using a preliminary version of the MASSA algorithm^56^ implemented in the KNIME Analytics Platform^57^ and its RDKit nodes^58^ according to previous works following the protocol described in.^59–61^ The experimental enzymatic reactivity was codified as 0 (unreactive) and 1 (reactive).

The molecular fingerprints Fingerprinter, Extended Fingerprinter, EState, Graph Only, MACCS, PubChem, Substructure, Substructure Count, Klekota Roth, Klekota Roth Count, Atom Pair 2D, and Atom Pair 2D Count were calculated with PaDEL descriptors software.^62^ All fingerprint sets were used to build and validate the decision tree classification models in the KNIME Analytics Platform. These models were constructed by using the training data and varying the minimum number of samples per node from 2 to 20 in even numbers.

The model’s validation was performed using a 5-fold cross-validation and external validation. All generated models were confirmed by the Matthews correlation coefficient (MCC), F1-Score, area under the receiver operating characteristic curve (AUC), and true positive rate (TPR). The models were ranked primarily according to the external MCC (_ext_MCC), calculated from the test sample, and cross-validation MCC (_cv_MCC), calculated from the 5-fold cross-validation process. The chosen model was interpreted following the OECD principles for QSAR.^63^ Also, this model was submitted to the X-scramble validation by generating 100 models with X-variables scrambled,^64^ the leave-N-out validation in triplicate by varying the number of samples in the validation group from 5 to 40 in step 5, and leave-one-out validation.^65^ Its applicability domain was assessed by employing the bounding box approach^66^ with the principal component analysis (PCA) and distance consensus (Euclidean, Manhattan, cosine, and Wasserstein—probability distribution) using a threshold of 95% as described before.^67^

### Fukui Functions from DFT Calculations

Geometry optimization calculations for the selected carbonyl compounds were carried out using density functional theory, DFT, on software Gaussian 09 ^68^ using the functional B3LYP,^69^ the 6-31G­(d,p) basis set.^70,71^ Solvation effects were included through single-point calculations on the optimized gas-phase structures, using a polarizable continuum model (PCM),^72^ with water dielectric constant.^73^ The electronic charges were characterized utilizing the CHELPG method.^74^ Local orbitals were determined by employing the natural bond orbital (NBO) method; finite difference approximation and condensed Fukui functions were utilized for their acquisition. For this, a cubic grid was designated with an evenly spaced mesh, featuring a grid of one point every 0.3 Å. This methodology ensured comprehensive sampling of points within the range of 0 to 2.8 Å around each atom, thus encompassing their van der Waals radii.^68^ The Fukui function estimates were obtained using the software UCA_Fukui.^75^ The Fukui functions for the carbonyl group *f* ^+^ and *f* ^–^, associated with the electrophilicity and nucleophilicity, respectively, were calculated using the following equations:^76,77^

$$f^{+}\left(r\right)={(\frac{\partial \rho \left(r\right)}{\partial N})}_{v(r)}^{+}=\rho_{N+1}\left(r\right)-\rho_{N}\left(r\right)$$

$$f^{-}\left(r\right)={(\frac{\partial \rho \left(r\right)}{\partial N})}_{v(r)}^{-}=\rho_{N}\left(r\right)-\rho_{N-1}\left(r\right)$$

where ρ_*N*_(*r*) is the electron density at coordinate *r*, *v*(*r*) is the external potential, and *N* is the number of electrons. The dual Fukui descriptor, *f* ^(2)^, is a second-order descriptor obtained from the Fukui function *f*(*r*) and described by the following equation:

$$f^{(2)}\left(r\right)={(\frac{\partial f\left(r\right)}{\partial N})}_{v(r)}=f^{+}\left(r\right)-f^{-}\left(r\right)$$

In the finite difference approximation, *f* ^(2)^ stands for the difference between carbonyl group electrophilicity (*f* ^+^) and nucleophilicity (*f* ^–^). The dual descriptor is able to simultaneously predict specific sites of nucleophilic (*f* ^(2)^ > 0) and electrophilic (*f* ^(2)^ < 0) attacks.

To visualize the dual descriptor’s topology on the structure of selected molecules, we used the software Multiwfn.^78^ This software takes the Fukui functions, calculated with the finite difference approximation, as input and performs the frontier molecular orbital approximation, FMO, to generate the density electronic isosurface.^79^

### Molecular Docking

The *Tf*NCS-dopamine-aldehyde-ketone complexes were modeled through molecular docking simulations. The crystal structure of *Tf*NCS cocrystallized with a transition-state mimicking compound 4-[2-[2-(4-methoxyphenyl)­ethylamine]­ethyl]­benzene-1,2-diol (pdb id: 5NON) was used in the docking simulations.^22^ The pdb file was edited as follows: (i) *Tf*NCS pdb file chains B and C were deleted to keep *Tf*NCS as a monomer as identified experimentally in solution;^34,80^ (ii) *Tf*NCS pdb file chain A was edited by manually removing Glu107 amino acid side-chain minor conformation and filling atom gaps in the pdb file using the server MolProbity;^81^ (iii) the coordinates and conformation of the dopamine substrate were manually modeled from the cocrystallized transition-state mimicking compound. The docking simulations were carried out with the software GOLD using the Gold Score as a scoring function, and the genetic algorithm with 200% efficiency was set up to generate 100 poses per ligand.^82^ The edited *Tf*NCS crystal structure bound to the modeled dopaminium cation was used as the target for the aldehydes and ketones, and the dopaminium nitrogen was defined as the center of the *Tf*NCS-dopamine binding site to direct the molecular docking simulations. The interactions were identified using the software Discovery Studio.^83^

### Molecular Dynamics

MD simulations were performed with the AMBER14 software,^84^ using the ff14SB force field^85^ for *Tf*NCS, TIP3P^86^ for the water molecules, and the General Amber Force Field, GAFF,^87^ for the *Tf*NCS-substrate candidates. The ANTECHAMBER module^88^ was used to check and correct carbonyl compounds’ missing parameters. The AM1-bcc charge model^89,90^ was applied for atomic charge point calculations. The first enzyme–substrate complex structures were solvated in a rectangular box with minimal distances of 10 Å between the box edges and any atom. Na^+^ ions were added to neutralize the system’s net charge. The minimization process was performed in two steps: the first minimization was performed with 5000 steps, 1000 using the steepest descendent algorithm^91^ and 4000 using the conjugate gradient with a restriction potential of 100 kcal mol^–1^Å^–2^.^92,93^ After that, a second minimization process was performed with the same protocol and number of steps but with no imposed restrictions.^94^

The systems were heated from 5 to 300 K in an NVT ensemble, alternating between heating and equilibration in 6 steps until the final temperature. Long-range and electrostatic interactions were treated with the particle mesh Ewald (PME) method.^95^ In the production step, the systems were simulated in an NPT ensemble, at 300 K and 1 bar, under periodic boundary conditions and regulated by the Berendsen isotopic barostat.^96^ The NPT production step of 100 ns was simulated for all molecular dynamics. The coordinates were saved at 10 ps. The MD simulations were performed using a 6-processor workstation Intel^Ⓡ^ Core i7–6800K, 3.60 GHz base frequency, 16 GB RAM, and NVIDIA GeForce GTX 1070 graphic cards. All MD simulations were analyzed using the cpptraj^97^ and the parmed^84^ packages, the software VMD v.1.9.3,^98^ and python3 algorithms. For each ternary complex, three independent replicas were produced and analyzed.

## Results and Discussion

### Determination of Reactivity of Aldehydes toward the *Tf*NCS-Catalyzed Reaction by NMR

NMR spectroscopy was used to evaluate the reactivity of four aldehydes in the *Tf*NCS-catalyzed Pictet–Spengler reaction with dopamine. The biocatalyzed reaction of hexanal and dopamine monitored by ^1^H NMR revealed two new singlets with chemical shift values of 6.74 and 6.76 ppm, which were assigned as the reaction product’s H3′ and H4’ (Figure 2) based on NMR spectra analysis and by comparing with previously published data.^26^ The decay of ^1^H NMR signals of dopamine aromatic H3 and H4 and the increment of the reaction product H3′ and H4’ signals were used to follow the reaction.^99^ Similarly, two singlets between 6.5 and 7.0 ppm are also seen in the reaction product obtained from the dopamine and octanal (Figure S03), but not in the control reaction (in the absence of the enzyme), thus corroborating with product formation through the *Tf*NCS catalyzed Pictet–Spengler reaction. Reactions of dopamine with 2-naphthaldehyde, piperonal, and terephthalaldehyde were also monitored by NMR but no product was detected after 24h, so these aldehydes were classified as “unreactive”. The chemical structures of the four aldehydes were incorporated into the library of *Tf*NCS active and inactive carbonyl compounds.

**Figure 2:**
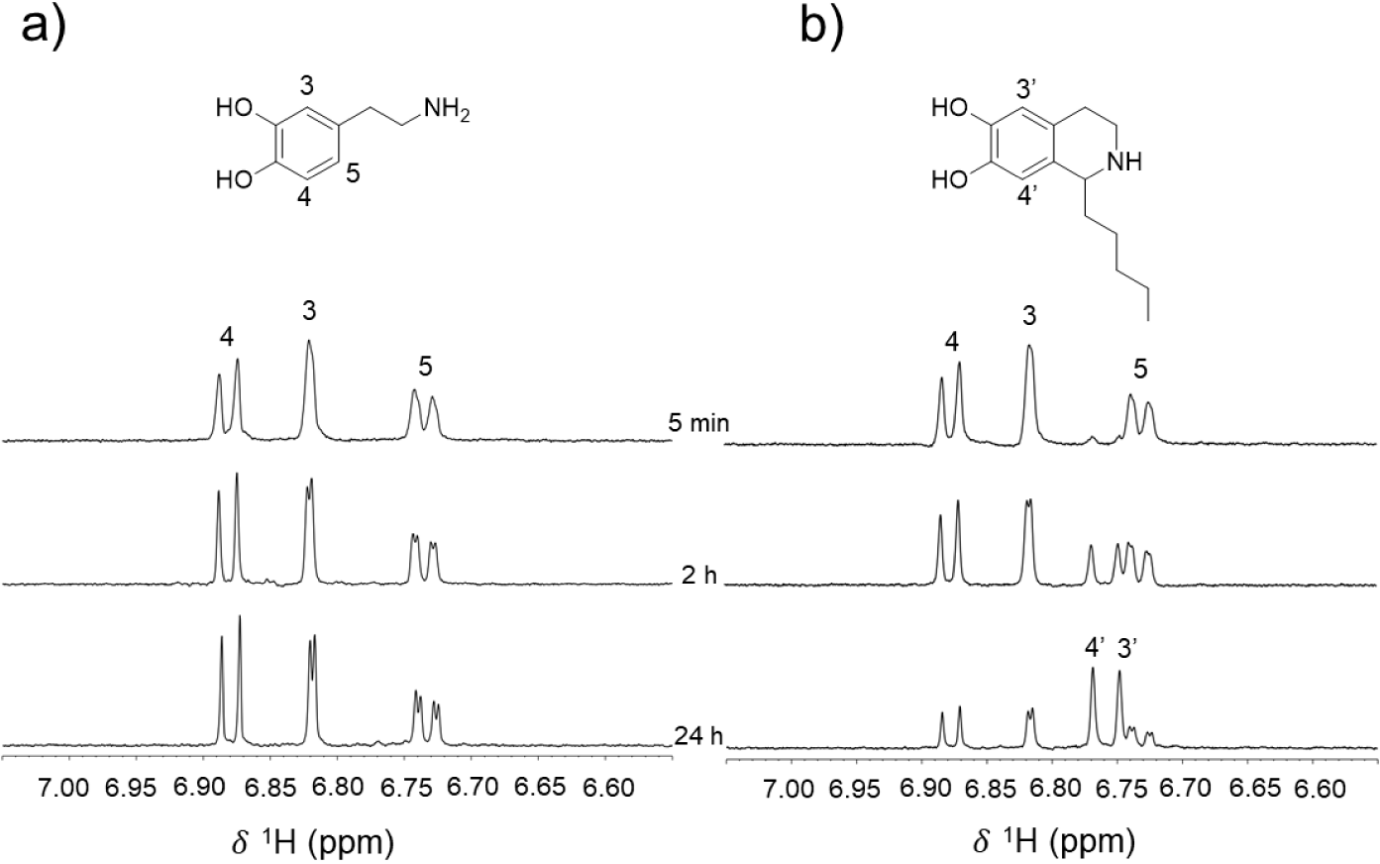
Monitoring of *Tf*NCS-catalyzed reaction of hexanal with dopamine by NMR spectroscopy: (a) aromatic region of 1D ^1^H NMR spectra of control solutions without the *Tf*NCS aliquot over the same time window used for the *Tf*NCS-catalyzed reaction; (b) 1D ^1^H NMR spectra of the *Tf*NCS-catalyzed reaction between dopamine and hexanal monitored at 5 min, 2 h, and 24 h after the *Tf*NCS addition. The reaction was performed in 50 mM HEPES buffer solution, 5 mM ascorbic acid, 2.5 mM aldehyde, 2.5 mM dopamine, 10% v/v DMSO, 10% v/v D_2_O, and 0.139 mM DSS, as an internal NMR standard, at 37 °C.

### QSAR Model

The chemical structures of 53 carbonyl compounds (49 from literature and 4 from the NMR experiments) were organized into two groups: the training and test set aiming to separate the carbonyl compounds in reactive and unreactive according to their reactivity in *Tf*NCS-catalyzed reaction assays and to identify chemical properties related to their reactivity and lack of reactivity in this biocatalyzed reaction. The training set of chemical structures was used to construct the model, while the “test one” was used to evaluate the models’ predictive performance for different molecules; 120 decision tree classifiers were built. The MCC value was used as the main metric to identify the best models since this methodology can summarize all attributes of a confusion matrix in a single index value.^100,101^ Models with MCC values higher than zero are considered predictive, and those with MCC values of 1 are perfect models. The model quality for each fingerprint was evaluated with MCC values by using 5-fold cross-validation (_cv_MCC) and external validation (_ext_MCC). The values of _cv_MCC and _ext_MCC are shown in Figure 3a. The model constructed with the MACCS fingerprint showed the higher _cv_MCC (0.704) and _ext_MCC (0.828) using six molecules as the smallest sample number in the node. MACCS also outperformed the other fingerprints when the externally and cross-validated AUC in the ROC curve was used as a metric (Figure 3b). The ROC curve evaluates simultaneously the true positive rate and the false positive rate distributions, and AUC values above 0.5 represent the degree of discrimination between these two distributions.^102^ To ensure that the model was not obtained by chance, the X-scrambling validation was performed. The scrambled models failed in internal and/or external validation compared to the original model, reinforcing that models generated with random data are poorly predictive and/or robust, and the used variables strongly correlate with the catalytic activity (Figure 3c). Additionaly, the leave-N-out validation demonstrated the model’s robustness across different sampling sizes during cros-validation, suggesting that the changes in data set size and composition do not affect the model’s robustness (Figure 3d). A PCA was conducted on the compounds in the training set using the MACCS fingerprint. The applicability domain of the training set was evaluated by projecting the test set compounds onto the PCA eigenvectors and then calculating the distance between the positions of the training and test compounds. The results showed that all test set compounds were within the applicability domain.

**Figure 3:**
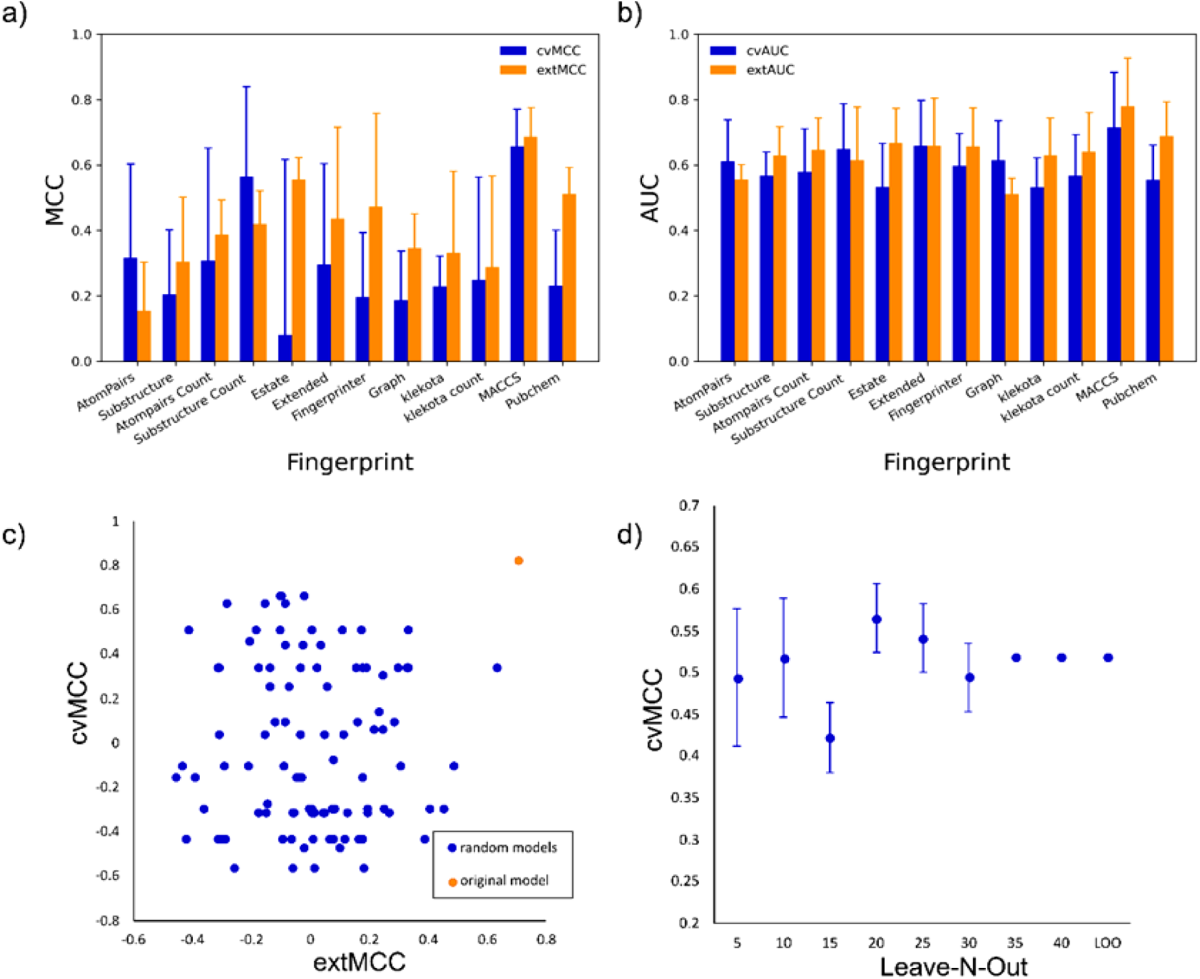
Validation metrics for the decision tree models. (a) Mean value of external validation MCC (extMCC) and cross-validation MCC (cvMCC); (b) mean value of external validation AUC (extAUC) and cross-validation AUC (cvAUC); (c) comparison between MCC values for external validation (extMCC) and cross-validation (cvMCC) for the original (orange) and scrambled (blue) models; (d) cross-validation MCC (cvMCC) for leave-N-out validation, including leave-one-out (LOO) validation.

The decision tree analysis can supply the chemical interpretation of the features used to predict the carbonyl compounds’ activity. The most predictable decision tree using the MACCS fingerprint is shown in Figure 4.

**Figure 4:**
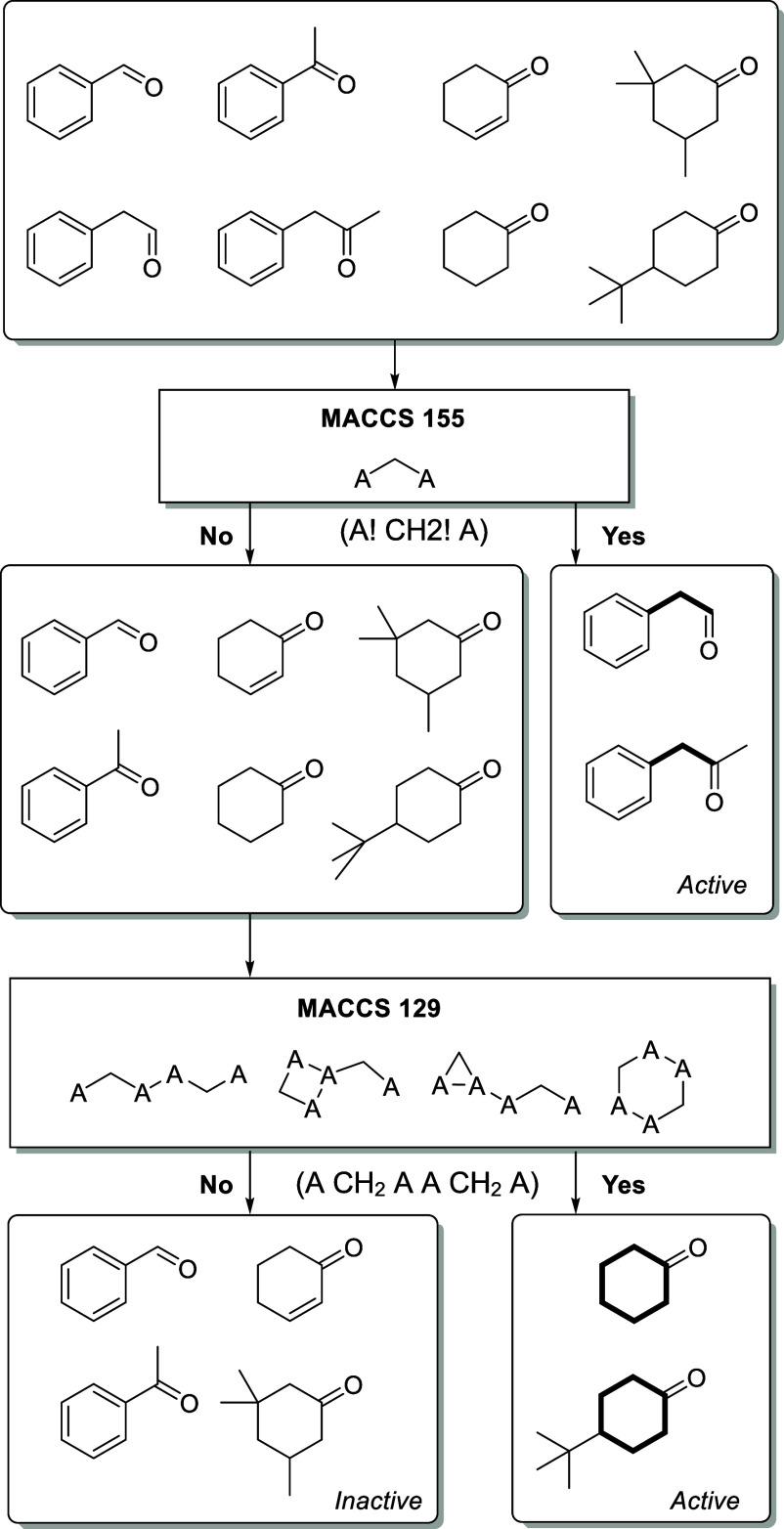
Graphical representation of the most predictable decision tree analysis using the MACCS fingerprint. The symbol “!” in A and CH_2_ means a noncyclic group.

The MACCS155 feature was used in the first split in the decision tree (Figure 4). Molecules with the MACCS155 feature were classified as active. MACCS155 can be chemically interpreted as a methylene group between two atoms (A! CH2! A), where “!” means a noncyclic atom. The two “A”s refers to carbons, with one of them mandatorily a carbonyl carbon. A second split was needed for the molecules without this feature, this time using the MACCS129. Molecules with this feature were considered active, and molecules without it were inactive. On both sides, MACCS129 stands for two methylene groups separated by two atoms (A CH_2_ A A CH_2_ A) and bound to one more (Figure 4). The QSAR highlighted the importance of the methylene group near the carbonyl group in classifying a compound as active in the *Tf*NCS-catalyzed Pictet–Spengler reaction.

However, it is important to mention that the activity of the wild-type *Tf*NCS toward alpha-substituted aldehydes^18,20,21,103^ as well as benzaldehydes^19^ has been recently reported. Although these findings may seem to disagree with our model in a first analysis, it might be noted that they relied on experiments performed with relatively high substrate loads to overcome high dissociation constants (*K*_d_) values for dopamine and aldehyde showed by them.^18,80^ Indeed, reported *Tf*NCS-catalyzed reactions with dopamine and alpha-substituted aldehydes to give enantioenriched tetrahydroquinolines in good yields were performed with a 20 mM concentration of the carbonyl substrate.^20^ In contrast, all aldehydes selected for our library were classified as unreactive based on assays employing 1 to 5 mM aldehyde concentrations (see the Supporting Information). For these reasons, the Results and Discussion herein must be critically and carefully interpreted as possibly reflecting differences in *K*_M_ values and in the reactivity of the substrate candidates instead of being predictive of “prohibited” substrates.

### Local Electronic Properties by Fukui Functions

The QSAR modeling shows that aldehydes and ketones with the unconjugated carbonyl group might be classified as a *Tf*NCS subtract, i.e., as “reactive” carbonyl compounds. Because conjugation implies not only steric but also electronic effects on the carbonyl group, we decided to investigate whether the electronic properties of the carbonyl groups could correlate with the reactivity of carbonyl compounds in the *Tf*NCS-catalyzed reaction. We then investigated the electronic distribution of a series of carbonyl compounds in the *Tf*NCS-catalyzed Pictet–Spengler reaction by Fukui function, *f* ^+^, which is useful to estimate the electrophilicity at distinct atoms, and the dual descriptor, a robust and efficient descriptor of both electrophilicity and nucleophilicity of amphipathic molecules.^76^ We decided to use the *f* ^+^ and the *f* ^(2)^ to compare the results of two different descriptors related to the electrophilicity at distinct atoms; one, a first-order descriptor and the other a second order one.^77^ The Fukui function *f* ^+^ and the dual descriptor *f* ^(2)^,calculated at the carbonyl carbon coordinate, are listed in Table S02. The Fukui function *f* ^+^ values, for each carbonyl compound, are plotted, in Figure 5a, as a function of the dual descriptor *f* ^(2)^. The *f* ^+^ is the electrophilicity descriptor, and *f* ^(2)^ describes the difference between electrophilicity and nucleophilicity at the used atomic position.^75^ The compounds that were experimentally identified as *Tf*NCS substrates are identified with blue circles; those in red are the experimentally inactive ones. From the *f* ^+^ vs *f* ^(2)^ plot, it is possible to separate the compounds into two main clusters: group 1 (red) and 2 (blue).

**Figure 5:**
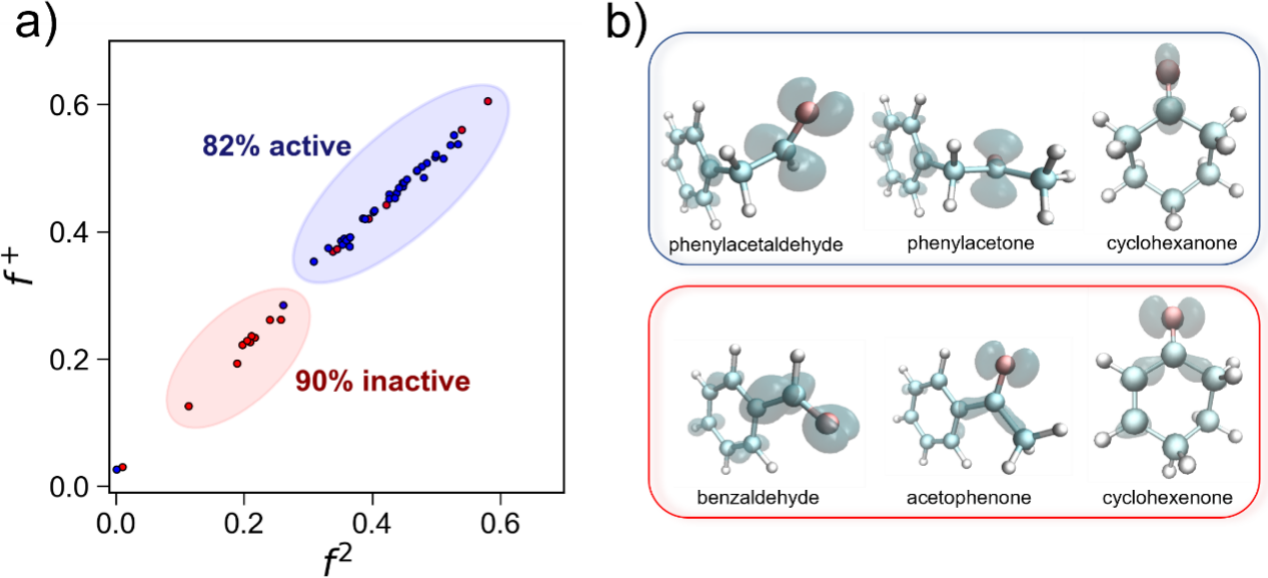
(a) Fukui function *f* ^+^ vs the dual descriptor *f* ^(2)^. The compounds experimentally classified as actives in the *Tf*NCS-catalyzed reaction are identified with blue cycles and those as inactives in red cycles; (b) electronic density isosurface of the dual descriptor *f* ^(2)^ on the chemical structure of representative compounds, shown in (a), of groups 1 (inactive; red) and 2 (active, blue). The topology of the dual descriptor *f* ^(2)^ was calculated with the software Multiwfn using the DFT output files, and the figures were generated using the software VMD.

Group 1 contains 10 compounds, of which only one has shown activity in this reaction, albeit with low conversion rates.^20^ In contrast, group 2 consists of 41 compounds, of which 34 (82%) are active in the *Tf*NCS-catalyzed reaction and 7 outliers (within the cluster but discrepant reactivity). Our results show that clustering based on Fukui functions at the carbonyl carbon position supplies insight into the reactivity of carbonyl compounds in the *Tf*NCS-catalyzed reaction. Specifically, compounds with low electrophilicity at the carbonyl carbon are less likely to undergo nucleophilic attack by dopamine’s amine nitrogen during the first step of the reaction. The Pictet–Spengler reaction proceeds via several steps, including an initial nucleophilic attack of dopamine nitrogen onto the carbonyl group followed by dehydration to give an iminium intermediate; cyclization takes part then through an aromatic electrophilic substitution, which encompasses a nucleophilic attack of the ring carbon onto the iminium electrophile followed by proton transfer resulting in rearomatization.^42^ Theoretical studies^42^ and evidence from kinetic isotope effects^104^ have supported rearomatization via proton transfer as the rate-limiting step of the *Tf*NCS-catalyzed Pictet–Spengler reaction of dopamine and 4-hydroxyphenylacetaldehyde. In contrast, the initial nucleophilic attack of nitrogen on the carbonyl group would be quite facile. Although it is not clear if this proposed mechanism also applies to other aldehydes and ketones, the correlation of electrophilicity with the reactivity of carbonyl compounds toward the *Tf*NCS-catalyzed reaction we found herein should not be misinterpreted as resulting directly from their higher reactivity toward the initial nucleophilic attack of dopamine nitrogen. A possible explanation for the observed correlation is that the electrophilicity of the carbonyl compounds and some properties favoring binding and reactivity of the derived reaction intermediates result from the same structural features.

### Molecular Docking and MD Simulations

We could identify a relation between the experimental reactivity of carbonyl compounds in *Tf*NCS-catalyzed reactions and the electronic properties of carbonyl carbons in aldehydes and ketones. Despite that, seven among 41 compounds of group 2 are unreactive in the *Tf*NCS-catalyzed reaction. The reasons for this discrepancy may rely on steric effects and other physical-chemical properties important for the outcome of the catalyzed Pictet–Spengler reaction, such as unfavorable contacts and high-energy barriers of intermediates and transition states. We investigated the presence of unfavorable contacts between the *Tf*NCS catalytic site and the experimentally unreactive carbonyl compounds within group 2. To continue this study, we organized the outliers in Figure 5a into two separated groups: group A, composed of aliphatic (one reactive and three unreactive compounds), and group B, consisting of cyclic carbonyl compounds (one reactive and four unreactive compounds; Figure 6a).

**Figure 6:**
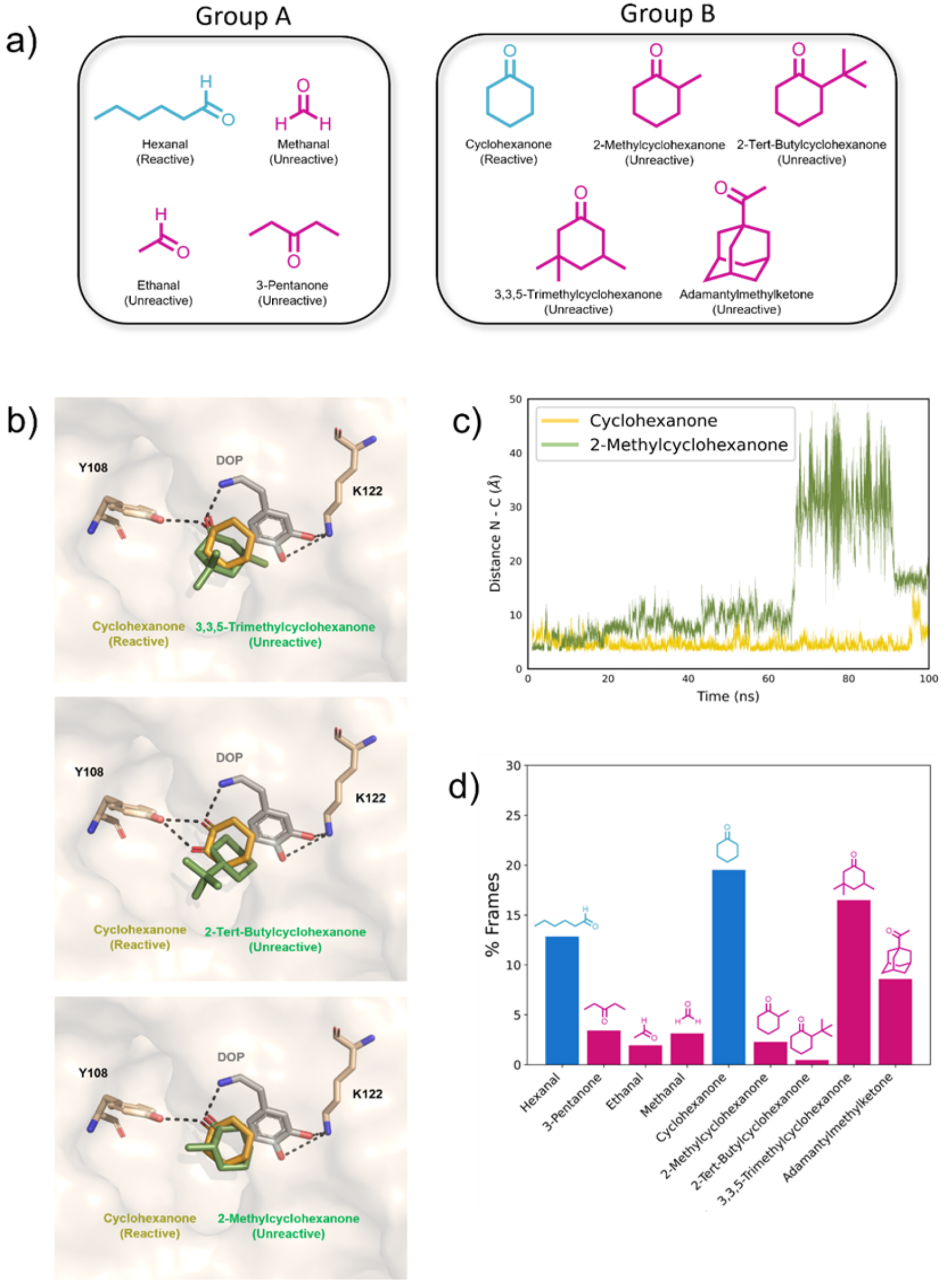
(a) Chemical structure of carbonyl compounds selected to study the interactions between *Tf*NCS and aldehydes/ketones classified as outliers in the Fukui description calculation and clustering methodology; (b) graphical representation of molecular docking simulation models highlighting the poses of cyclohexanone, 3,3,5-trimethylcyclohexanone, 2-methylcyclohexanone, and 2-*tert*-butylcyclohexanone bound to the *Tf*NCS catalytic site. The interactions between the ketones, dopamine, and residues Y108 and K122 are indicated; (c) distance N−C=O, as a function of MD simulation time: distance between the dopamine nitrogen and the carbonyl carbon of cyclohexanone (orange) and 2-methylcyclohexanone (green). MD simulations were conducted using the software Amber 14; (d) the relative number of frames where the distance between C (carbonyl) and N (dopamine) is shorter than or equal to 4.5 Å and obtained from three independent MD simulations of 100 ns each. Experimentally, reactive compounds are colored blue, and unreactive ones are magenta.

We performed molecular docking simulations to model the ternary complexes of *Tf*NCS-dopamine-carbonyl compounds and to identify important interactions between *Tf*NCS and aldehydes and ketones (Figures 6b and S04). The dopamine structure was correctly positioned in the *Tf*NCS catalytic cavity, interacting with residue K122 and with its amine group interacting with catalytic residues D141 and E110. We also obtained poses of cyclic and aliphatic aldehydes and ketones correctly positioned in the *Tf*NCS cavity. The acetaldehyde interacted only with residue Y108, suggesting a lower affinity with *Tf*NCS. The active compound, cyclohexanone, interacted with *Tf*NCS by polar interactions with Y108 and the dopamine amine group and through hydrophobic interactions with P179 and M183. A substituent on the α position of cyclic hexanones, such as in 2-methylcyclohexanone and 2-*tert*-butylcyclohexanone, increased the number of hydrophobic contacts with *Tf*NCS compared to cyclohexanone. The apolar interactions between 2-*tert*-butylcyclohexanone and *Tf*NCS hydrophobic pockets, formed by residues L76, F80, F99, and P179 (Figure S04), caused a significant change in the orientation of the carbonyl group to the amine group of dopamine (Figure 6b). This could diminish the probability of nucleophilic attack by the dopamine group at the carbonyl carbon of 2-*tert*-butylcyclohexanone and explain why this compound is inactive in *Tf*NCS-catalyzed reactions. Overall, molecular docking showed the importance of hydrophobic interactions and carbonyl group orientation in determining the carbonyl compounds’ reactivity in *Tf*NCS-catalyzed reactions.

We performed three MD simulation replicas for each ternary complex generated from the molecular docking study (Figure S04). To determine the stability of the complex between dopamine and aldehydes/ketones, we analyzed the distance between the carbonyl carbon and the amine nitrogen of dopamine during the simulation. A distance cut of 4.5 Å between dopamine nitrogen and the carbonyl compound was used as an indicator of the correct relative positioning of dopamine and aldehydes/ketones within the *Tf*NCS catalytic site.^20,105^ We counted the number of ideal frames for each compound during the MD simulation (Figures 6c,d, S05 and S06), and this was used as an indicator of the *Tf*NCS interactions with aldehydes/ketones.

*Tf*NCS–dopamine–carbonyl compound complexes with short chains such as ethanal and methanal showed less stability during the MD simulations (Figures 6d and S05), indicating lower affinity with *Tf*NCS. The 3-pentanone complex also showed lower stability (Figures 6d and S05) due to steric hindrance between the 3-pentanone methyl groups and *Tf*NCS residue side chains (Figure S04). Steric hindrance also affected the interaction of 2-methylcyclohexanone, 2-*tert*-butylcyclohexanone, and adamantyl ketone (Figures 6b–d and S06). Our results suggest that substitution on the β carbon of ketones, such as 3,3,5-trimethylcyclohexanone, is less critical than substitution on the α carbon, such as 2-*tert*-butylcyclohexanone. The reason for 3,3,5-trimethylcyclohexanone unreactivity may be associated with the energy of the tetrahedral intermediate or other intermediate states.

## Conclusion

In this work, we investigated the chemical signatures in aldehydes and ketones relevant to their reactivity toward the *Tf*NCS-catalyzed Pictet–Spengler reaction using molecular modeling strategies with scalable complexity. For this purpose, we organized a library of experimentally classified reactive and unreactive aldehydes and ketones in *Tf*NCS-catalyzed reactions. We applied QSAR and Fukui functions based on structure optimization by DFT to find the chemical properties that are important to predict the reactivity of carbonyl compounds. We discovered that the structure of the carbonyl compounds, including the electron density on the carbonyl group, presents a good correlation with the reactivity of ketones and aldehydes in the enzymatic Pictet–Spengler reaction. Aldehydes and ketones with low electrophilicity at the carbonyl carbon coordinate are more likely to be unreactive in *Tf*NCS-catalyzed reactions. Interestingly, this correlation was not observed for seven compounds. For those outlier compounds, molecular docking and molecular dynamic simulations were used to assess other critical physical-chemical properties. Aldehydes and ketones with short chains are more likely to escape the *Tf*NCS cavity due to lacking interactions. On the other hand, steric hindrance seems to destabilize the ternary complex with bulky carbonyl molecules. For cyclic ketones, we found that substitution on β carbon is less critical to the *Tf*NCS-catalyzed reaction than substitution on α carbon. This information is summarized in Figure 7. These findings, however, must be critically interpreted and applied to understand differences in the reactivity of carbonyl compounds toward the *Tf*NCS-catalyzed Pictet–Spengler reaction instead of being used to rule out acceptance of some compounds as substrate by wild-type *Tf*NCS. Together, our results show an example of the application of in silico techniques to understand enzyme promiscuity and specificity, with particular attention to the combination of machine learning methodologies, DFT electronic structure calculations, and molecular dynamic simulation.

**Figure 7:**
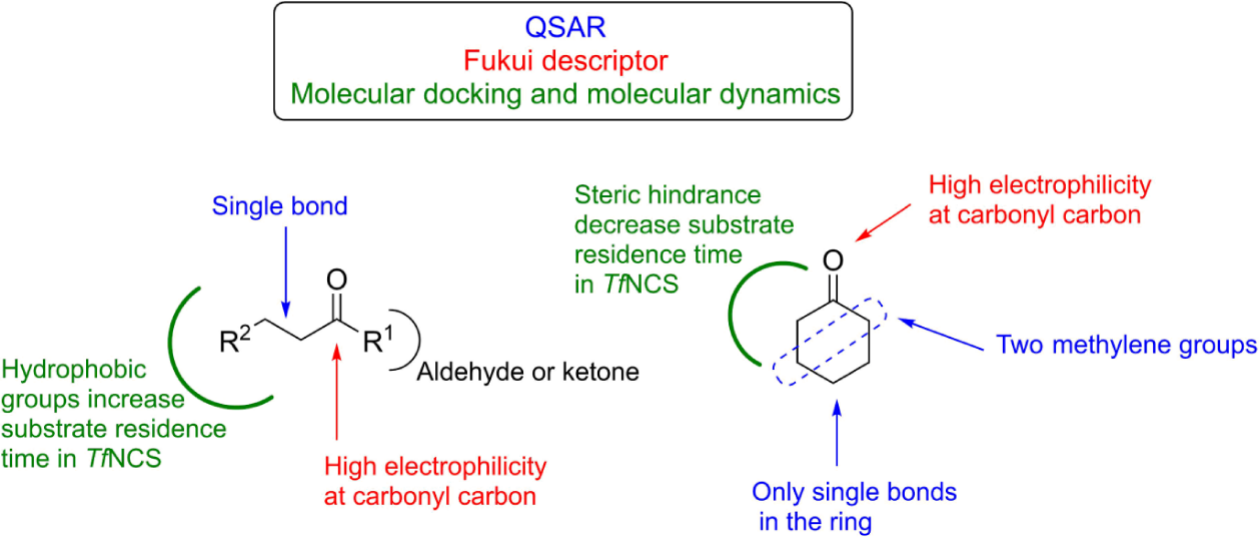
Scheme summarizing the properties of aldehydes and ketones important for their interaction with *Tf*NCS and *Tf*NCS activity as a catalyst in the Pictet–Spengler reaction with dopamine. The colors indicate the methodology used to identify highlighted properties.

## Supplementary Material





## Data Availability

The data associated with this work are accessible through a Zenodo repository (10.5281/zenodo.8432587), organized into three main folders: QSAR, Fukui, and MD. The QSAR folder contains molecular structures in the SDF format and their corresponding classes and training/split information in CSV files. Machine learning validation results and the descriptors used as input to train the discussed model are also available in this folder. The Fukui folder includes calculations of Fukui’s descriptors, with a separate fchk and log file for each molecule. Lastly, the MD folder encompasses molecular dynamics results and the associated archives from the MD production. A comprehensive description of each file can be found in the readme file, accessible in the Zenodo repository.

## Notes

The chemical structures of aldehydes and ketones composing the carbonyl compound library; description of the experimental methodology used to evaluate the *Tf*NCS activity for each compound of the organized library; results of *Tf*NCS enzymatic assay by NMR spectroscopy; table with aldehyde and ketone Fukui descriptors calculated at the carbonyl carbon coordinate of structure optimized by DFT, 2D interaction map of contacts between aldehydes and ketones bound to *Tf*NCS and dopamine, MD simulation results of the seven outlier compounds identified in the plot of *f* ^+^ vs *f* ^(2)^ (PDF)
SMILES representations of compounds (TXT)

## Abbreviations

AUC: area under curve
DFT: density functional theory
DMSO: dimethyl sulfoxide
MACCS: Molecular ACCess Systems keys fingerprint
MACCS129: MACCS’s fingerprint 129
MACCS155: MACCS’s fingerprint 155
MCC: Matthew correlation coefficient
NMR: nuclear magnetic resonance
NOESY: nuclear Overhauser effect spectroscopy
OECD: Organization for Economic Co-operation and Development
PCA: principal component analysis
QSAR: quantitative structure–activity relationship
ROC: receiver operating characteristics
*Tf*NCS,: (*S*)-NCS from
