# Supplementary material for: Understanding the Enzyme (S)‑Norcoclaurine Synthase Promiscuity to Aldehydes and Ketones
Source: J Chem Inf Model. 2024 May 22;64(11):4462–74. doi: 10.1021/acs.jcim.3c01773 (PMC12225969; doi:10.1021/acs.jcim.3c01773)
Supplement: Supplementary file 1 [file ci3c01773_si_001.pdf]

Supporting information

Understanding the enzyme (S)-Norcoclaurine Synthase promiscuity to aldehydes and ketones

Brunno A. Salvatti[1 ‡], Marcelo A. Chagas[2 ‡], Phillipe O. Fernandes[3 ‡], Yan F. X. Ladeira[1], Aline S. Bozzi[1], Veronica S. Valadares[4], Ana Paula Valente[5], Amanda S. de Miranda[1], Willian R. Rocha[1], Vinicius G. Maltarollo[3], and Adolfo H. Moraes[1].

[1]Departamento de Química, Instituto de Ciências Exatas, Universidade Federal de Minas Gerais, Belo Horizonte, Brazil, 31270-901; [2]Departamento de Ciências Exatas, Universidade do Estado de Minas Gerais, João Monlevade, Minas Gerais, Brazil, 35930-314. [3]Departamento de Produtos Farmacêuticos, Faculdade de Farmácia, Universidade Federal de Minas Gerais, Belo Horizonte, Brazil, 31270-901; [4]Departamento de Bioquímica e Imunologia, Instituto de Ciências Biológicas, Universidade Federal de Minas Gerais, Belo Horizonte, Brazil, 31270-901; [5]Centro Nacional de Ressonância Magnética Nuclear, Instituto de Bioquímica Médica Leopoldo de Meis, Centro de Ciências da Saúde, Universidade Federal do Rio de Janeiro, Rio de Janeiro, Brazil, 21.941-902.

Construction of the substance library

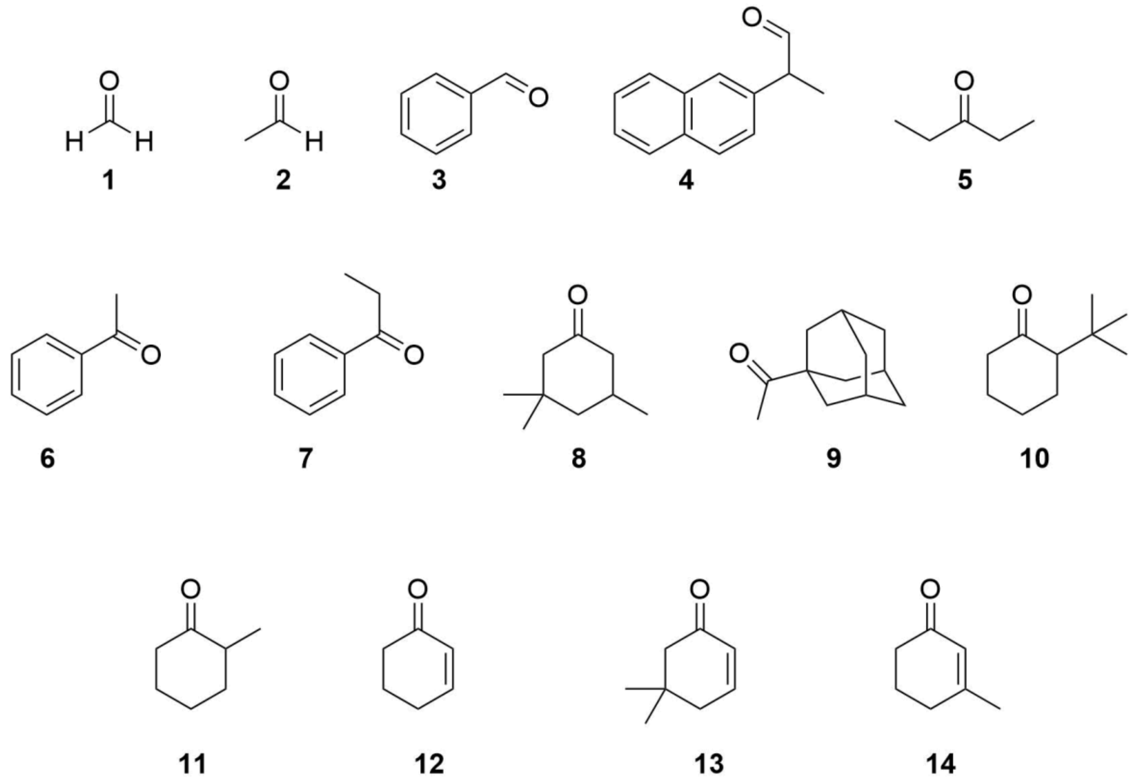

Supplementary Figure S01: Chemical structure of carbonyl compounds experimentally classified as unreactive in *Tf*NCS-catalyzed Pictet-Spengler reaction.

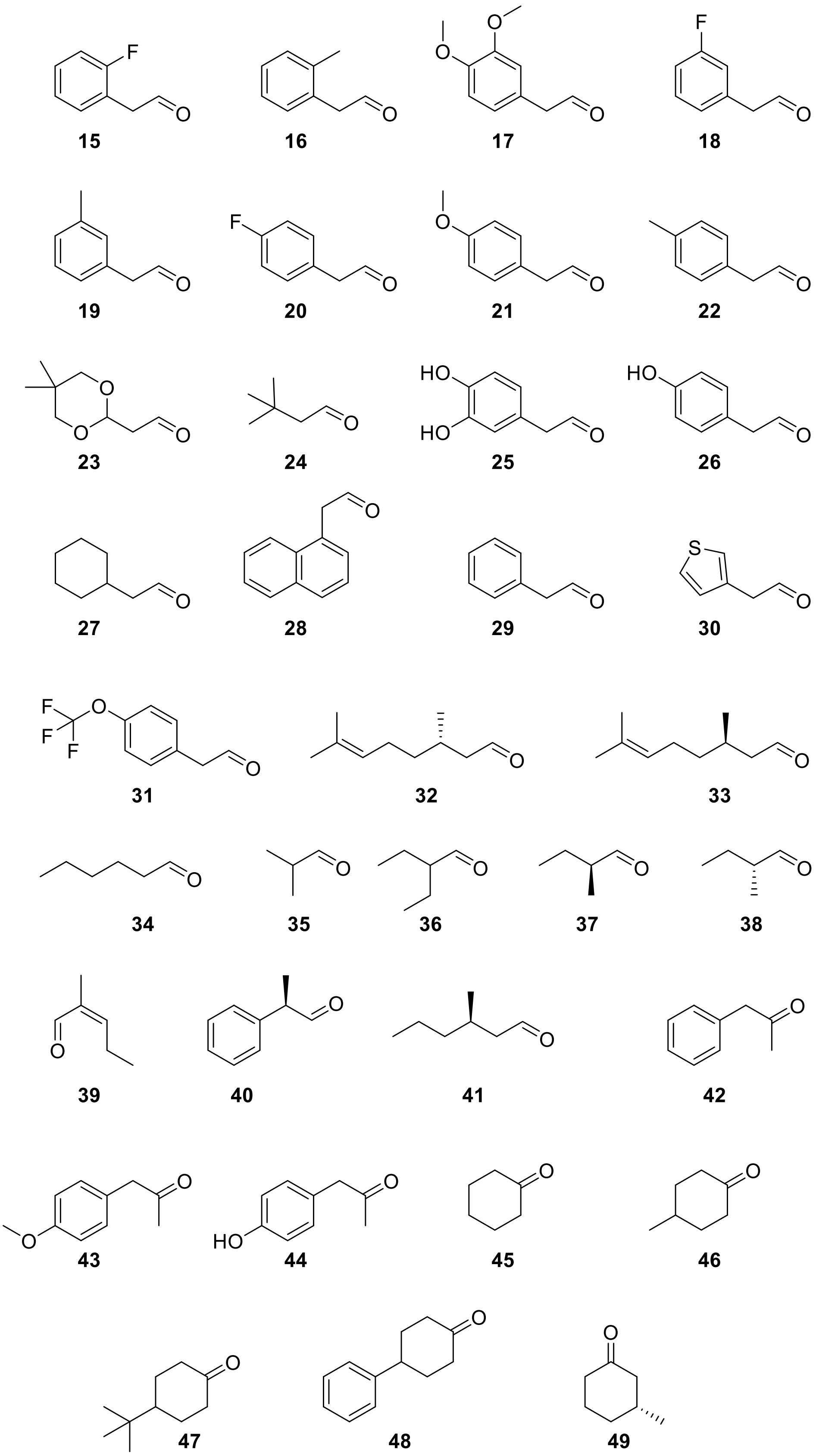

Supplementary Figure S02: Chemical structure of carbonyl compounds experimentally classified as reactive in *Tf*NCS-catalyzed Pictet-Spengler reaction.

Supplementary Table S01: Experimental methodology used for each compound tested in *Tf*NCS-catalyzed reaction assay. The numbers agree with Figures S01 and S02.

**Compounds**	**Methodology**
1 – 4 (unreactive)<br>15 – 31 (reactive)[1]	Dopamine (1 mM), Aldehyde (1 mM), *Tf*NCS (300 µM), TRIS buffer (100 mM, pH 7.0); room temperature; incubation time 1 hour; detection methods: LC-MS
32 – 34 (reactive) [2]	Varying dopamine concentration (500 µM - 20 mM) with constant aldehyde concentration (2.5 mM). Varying aldehyde concentrations (250 µM – 15 mM) with constant dopamine concentration (2.5 mM); temperature: 37 °C, incubation time: 30 s; detection method: HPLC.
35 – 41 (reactive) [3]	Dopamine (10 mM), Aldehyde (20 mM), Sodium ascorbate (5 mM), *Tf*NCS (0.2 mg / mL), HEPES buffer (100 mM, pH 7.5), DMSO (10% v/v), temperature 37°C, incubation time 18 h; detection method: HPLC
5 – 14 (unreactive)<br>42 – 49 (reactive) [4]	Dopamine (15 mM), Ketone (10 mM), Lysate (20% v/v), DMSO (10% v/v), temperature 37°C; incubation time 6h; detection method: HPLC

Enzyme activity monitored by NMR spectroscopy

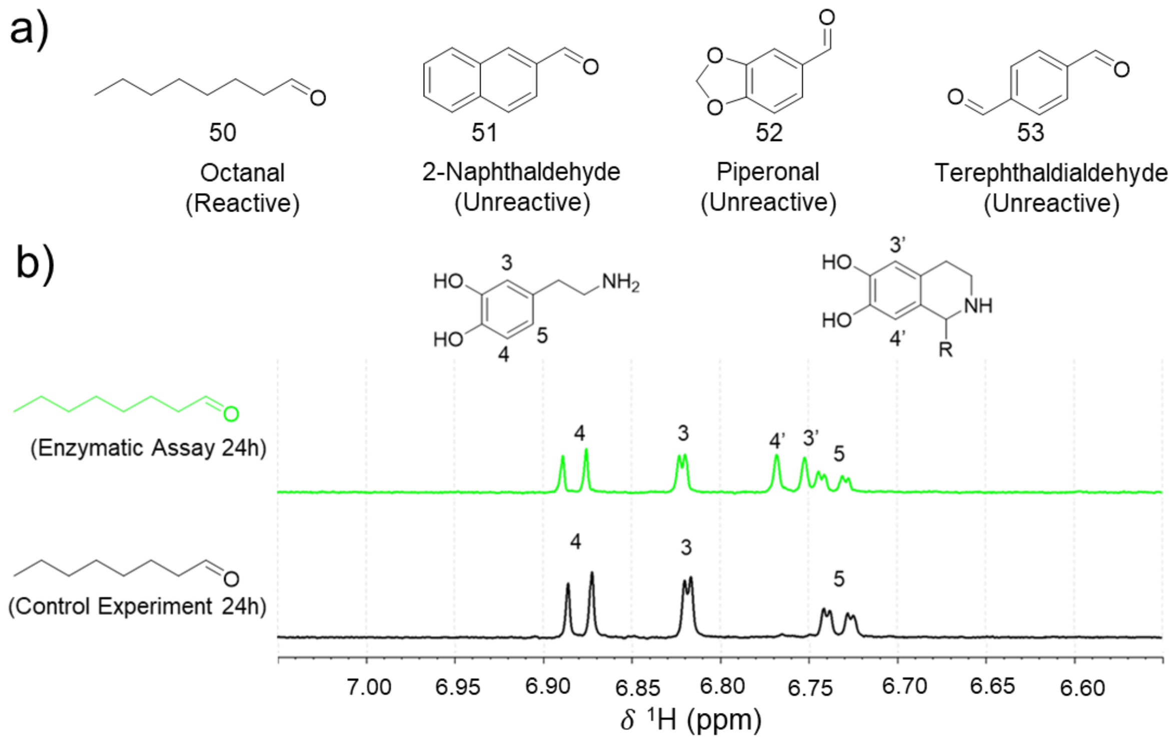

Supplementary Figure S03: *Tf*NCS enzymatic assay by NMR spectroscopy: a) Chemical structure of the evaluated aldehydes; b) ^{1}H 1D NMR spectra of dopamine aromatic region with the aromatic ^{1}H informed. The spectra were recorded after 24 hours after *Tf*NCS titration. The control experiment was performed without the *Tf*NCS addition. The reaction was performed in 50mM HEPES buffer solution, 5 mM ascorbic acid, 2.5 mM aldehyde, 2.5 mM dopamine, 10% v/v DMSO, 10% v/v D_2O, and 0.139 mM DSS, as internal NMR standard, at 37 °C. The ^{1}H NMR experiments were acquired using the ^{1}H solvent-presaturation NOESY sequence (Bruker code: noesypr1d) with the following acquisition parameters: number of scans = 128, number of acquisition points = 64k, recovery time = 30 s, and mixing time = 10 ms.

Calculated Fukui descriptors

Supplementary Table S02: Calculated Fukui descriptors for the carbonylic compounds using the structure optimized by DFT calculations.

Compound	Class	f+	f^2	Compound	Class	f+	f^2
**1**	Unreactive	0.605	0.5808	**28**	Reactive	0.0257	0.0012
**2**	Unreactive	0.5598	0.54	**29**	Reactive	0.4204	0.3857
**3**	Unreactive	0.261	0.2407	**30**	Reactive	0.4525	0.4356
**4**	Unreactive	0.0296	0.0104	**31**	Reactive	0.3853	0.3515
**5**	Unreactive	0.4419	0.4223	**32**	Reactive	0.4846	0.4808
**6**	Unreactive	0.2331	0.2173	**33**	Reactive	0.5374	0.5342
**7**	Unreactive	0.2259	0.2099	**34**	Reactive	0.5021	0.4777
**8**	Unreactive	0.3902	0.3643	**35**	Reactive	0.4963	0.4703
**9**	Unreactive	0.4199	0.3947	**36**	Reactive	0.4768	0.4479
**10**	Unreactive	0.3685	0.3386	**37**	Reactive	0.4769	0.4498
**11**	Unreactive	0.3722	0.3452	**38**	Reactive	0.4769	0.4498
**12**	Unreactive	0.2357	0.212	**39**	Reactive	0.2841	0.2611
**13**	Unreactive	0.2215	0.1978	**40**	Reactive	0.389	0.3564
**14**	Unreactive	0.2282	0.2052	**41**	Reactive	0.482	0.4544
**15**	Reactive	0.5359	0.5225	**42**	Reactive	0.3739	0.3315
**16**	Reactive	0.4584	0.4269	**43**	Reactive	0.4196	0.389
**17**	Reactive	0.4705	0.4482	**44**	Reactive	0.3529	0.3088
**18**	Reactive	0.5168	0.4987	**45**	Reactive	0,3868	0,3613
**19**	Reactive	0.4306	0.4021	**46**	Reactive	0.3793	0.3537
**20**	Reactive	0.5146	0.5114	**47**	Reactive	0.3861	0.3598
**21**	Reactive	0.4604	0.4385	**48**	Reactive	0.3762	0.3649
**22**	Reactive	0.433	0.4037	**49**	Reactive	0.3912	0.3656
**23**	Reactive	0.5076	0.4855	**50**	Reactive	0.5515	0.5279
**24**	Reactive	0.4684	0.4419	**51**	Unreactive	0.1926	0.1895
**25**	Reactive	0.5208	0.4997	**52**	Unreactive	0.2613	0.2575
**26**	Reactive	0.4513	0.4268	**53**	Unreactive	0.1253	0.1137
**27**	Reactive	0.4953	0.4699				

Interactions between the ligands and Tf**NCS *observed in the molecular docking***

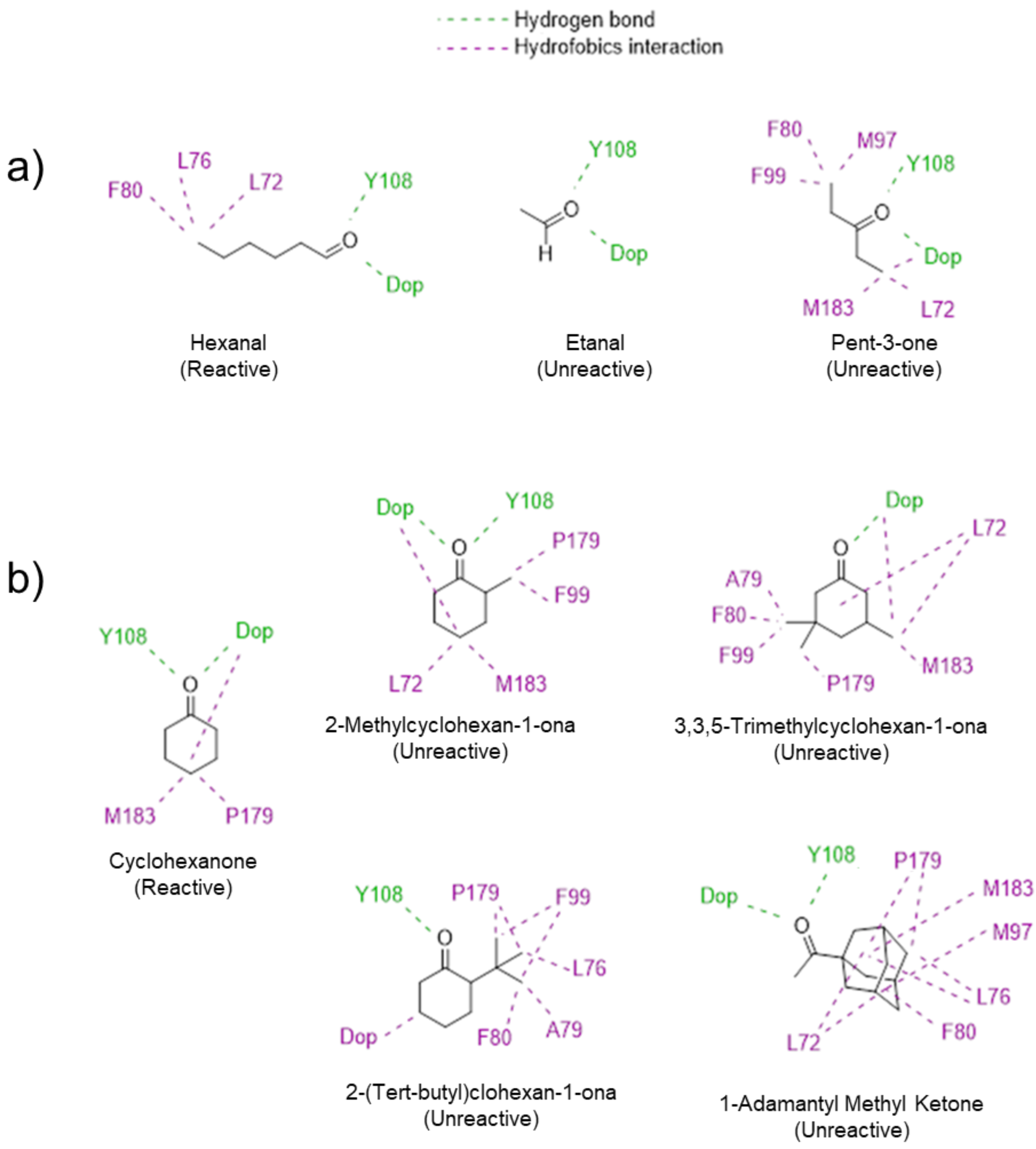

Supplementary Figure S04: 2D interaction map of contacts between aldehydes and ketones bound to *Tf*NCS and dopamine. a) aliphatic compounds; and b) cyclic compounds. The maps were elaborated based on interaction contact mapping performed with the software Discovery Studio. The ternary models, *Tf*NCS-dopamine-aldehyde/ketones were modeled by molecular docking simulations with the software Gold. The aldehydes and ketones coordinates with carbonyl groups positioned nearby the dopamine amine group were selected to mimic the nucleophilic attack.

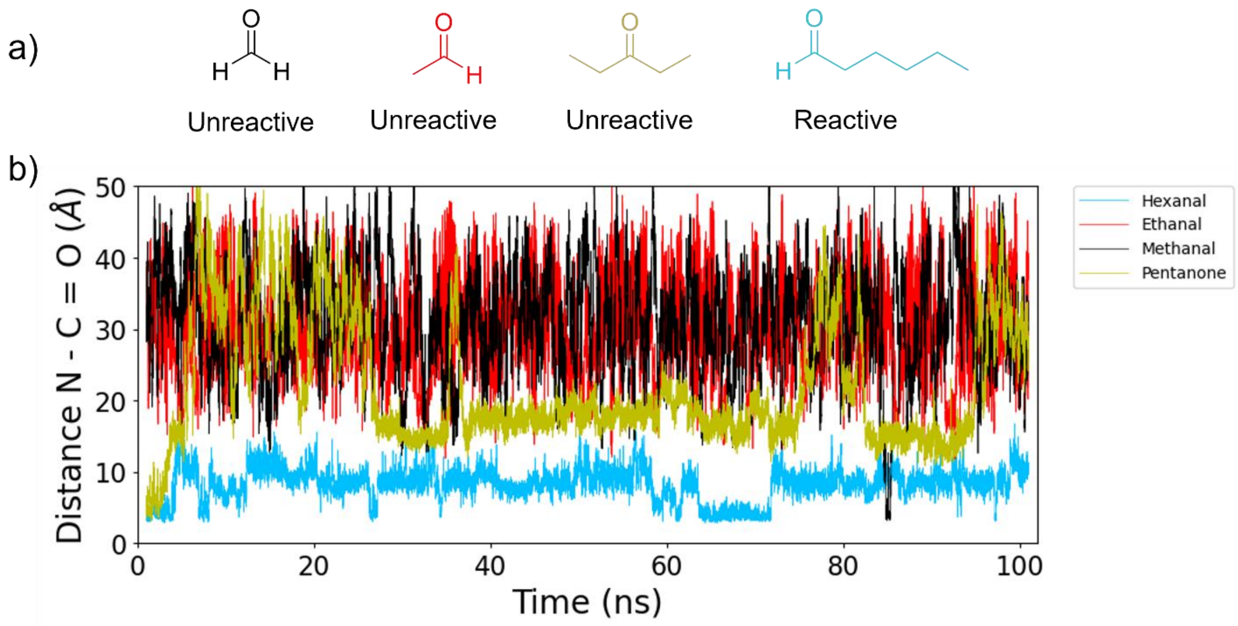

Supplementary Figure S05: Representative MD simulation of selected linear compounds. a) Chemichal structure of selected compounds; and b) distance between the dopamine nitrogen and the carbonyl carbon of group A aldehydes/ketones (Distance N – C=O) as a function of MD simulation time. MD simulations were carried out using the software Amber 14.

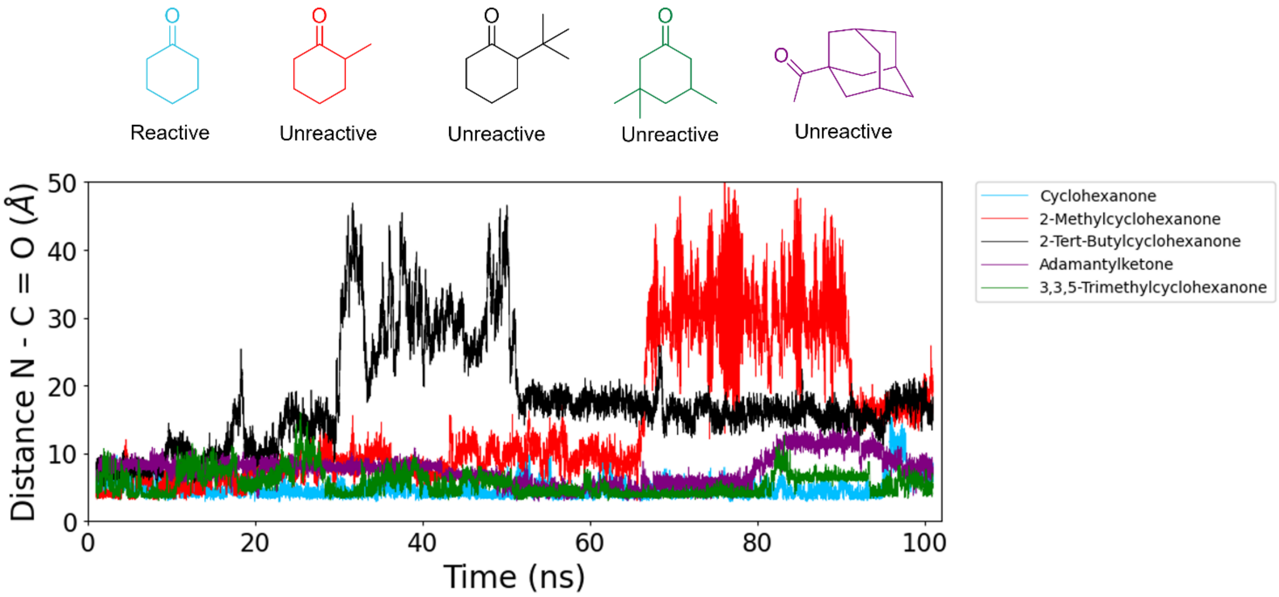

Supplementary Figure S06: Representative MD simulation of selected cyclic compounds. a) Chemichal structure of selected compounds; b) and distance between the dopamine nitrogen and the carbonyl carbon of group B aldehydes/ketones (Distance N – C=O) as a function of MD simulation time. MD simulations were carried out using the software Amber 14.

REFERENCES

(1) Ruff, B. M.; Bräse, S.; O'Connor, S. E. Biocatalytic Production of Tetrahydroisoquinolines. *Tetrahedron Lett.* **2012**, *53* (9), 1071–1074. https://doi.org/10.1016/j.tetlet.2011.12.089.

(2) Lichman, B. R.; Gershater, M. C.; Lamming, E. D.; Pesnot, T.; Sula, A.; Keep, N. H.; Hailes, H. C.; Ward, J. M. Dopamine-First Mechanism Enables the Rational Engineering of the Norcoclaurine Synthase Aldehyde Activity Profile. *FEBS J.* **2015**, *282* (6), 1137–1151. https://doi.org/10.1111/febs.13208.

(3) Roddan, R.; Gygli, G.; Sula, A.; Méndez-Sánchez, D.; Pleiss, J.; Ward, J. M.; Keep, N. H.; Hailes, H. C. Acceptance and Kinetic Resolution of α-Methyl-Substituted Aldehydes by Norcoclaurine Synthases. *ACS Catal.* **2019**, *9* (10), 9640–9649. https://doi.org/10.1021/acscatal.9b02699.

(4) Lichman, B. R.; Zhao, J.; Hailes, H. C.; Ward, J. M. Enzyme Catalysed Pictet-Spengler Formation of Chiral 1,1'-Disubstituted- A Nd Spiro-Tetrahydroisoquinolines. *Nat. Commun.* **2017**, *8*, 1–9. https://doi.org/10.1038/ncomms14883.
